# Studies in Mental Ineffic Ency

**Published:** 1920-04-17

**Authors:** 


### "STUDIES IN MENTAL INEFFIC ENCY."

The appearance of this periodical, issued by the Central Association for the Care of the Mentally Defective, was most welcome. The public mind is curiously ignorant of the questions and difficulties at issue in connection with that section of the community classed as mentally defective. Social workers, teachers, and others who come into contact with the cases experience a sense of helplessness, and often hopelessness, which more knowledge and interchange of idea might help in great part to dispel. The outlook is, or should be, brighter than ever before. Modern psychological research will have far-reaching results. Psychologists are applying their knowledge in many new fields with success, but it is very specially in the fields of mental pathology and education that their work lies. Much experience has been gained during the war, and the Freudian theories are being tested and examined in the light of the analysis of emotion and instinct which has been given us by our English psychologists. A great deal remains to be learnt and discovered in the care and treatment of those suffering from mental defect. Public interest needs rousing; the doctor, the social worker, and the teacher need some means of communication for up-to-date reliable information and for interchange of ideas.

### Meeting a Need.

We believe that "Studies in Mental Inefficiency" will meet that need, by bringing together and helping those already interested in the matter, and by public discussion rousing public interest, which is essential if legislative and educative reforms are to be carried through. It should be generally known, for example, that there is at the present time school accommodation for only one-half the mentally defective children in the country. Even were there sufficient accommodation much still remains to be done in finding further and better ways of educating this type of child. One aspect of this problem is touched upon by Miss Lucy Fildes in this first issue of the periodical under discussion in her article on "Individual Studies in the Light of their Educational Significance." The dull and backward child is a perpetual source of anxiety to the teacher, and it is well that we should be reminded that a method of instruction suitable for one child may not necessarily be suitable for another. The child's dulness may consist in not being able to conform to that particular method in use. A child unable to memorise through visual imagery may be quite capable of doing so by means of auditory or motor images. Typification has its dangers, but Miss Fildes pleads for the individual study of the best way in which a child can learn, and for recognition that the means by which a child learns is just as important a part of his report to be handed on as what he has learnt. The abnormal child requires careful study on the part of the teacher from the psychological standpoint.

### The Moral Imbecile.

Dr. Thredgold supplies an interesting article on "The Moral Imbecile," whom it has been very difficult to certify without proof of defect in ordinary intelligence. He is concerned with analysing very clearly, for his readers, the different points contained in the official statutory definition of a moral imbecile, and with showing that, by a careful and studied interpretation of the clauses, many of the difficulties experienced should be overcome. As social workers, doctors and others are aware many people belonging to this class of mental defective are by no means lacking in ordinary intelligence, the defect of their mind lying simply in a lack of any moral or social sense. If the defect has been permanent and has been evident from an early age, and the subject be undeterred by punishment, certification should be possible. One wonders whether the definition may be modified in future in the light of further psychological research, especially in regard to the phrase "from an early age," for, as Dr. Thredgold points out, "in some true moral imbeciles misconduct sufficient to attract attention may not occur until after school years." Until a subject be thrown upon his own responsibility in the world his particular tendencies to immoral conduct may not be excited. The moral imbecile is obviously a grave danger to society, and a very full understanding of the type and of the methods by which it may be treated is necessary. Very careful and skilled supervision is called for in the interest of the moral defective himself.

### Discussions and Information Provided.

There are countless problems in connection with mental deficiency other than the two raised in this issue, all of which demand study and discussion and interchange of opinion and experience. "Studies in Mental Inefficiency" will be fulfilling a really useful function to-day if it makes this possible through the medium of its pages. Dr. C. S. Myers, in speaking of disorders of the mind in his little book on the "Applications of Psychology," says "the difficulties are enhanced . . . by the popular ignorance and prejudice existing in regard to disorders of the mind." Public instruction and education is necessary if this problem of mental deficiency is to be dealt with effectively. It is one of the most important and pressing matters in any scheme of social reform to-day, and we much hope that this new periodical will fulfil its promise of stimulating interest in the question by promoting discussion and providing up-to-date information.

